# Integrating traditional medicine into the Ghanaian health system: perceptions and experiences of traditional medicine practitioners in the Ashanti region

**DOI:** 10.1093/inthealth/ihac059

**Published:** 2022-09-05

**Authors:** Irene G Ampomah, Bunmi S Malau-Aduli, Abdul-Aziz Seidu, Aduli E O Malau-Aduli, Theophilus I Emeto

**Affiliations:** College of Public Health, Medical and Veterinary Sciences, James Cook University, Townsville, QLD 4811, Australia; Department of Population and Health, University of Cape Coast, Cape Coast, Post Office Box UC 182, Ghana; College of Medicine and Dentistry, James Cook University, Townsville, QLD 4811, Australia; College of Public Health, Medical and Veterinary Sciences, James Cook University, Townsville, QLD 4811, Australia; Department of Population and Health, University of Cape Coast, Cape Coast, Post Office Box UC 182, Ghana; College of Public Health, Medical and Veterinary Sciences, James Cook University, Townsville, QLD 4811, Australia; College of Public Health, Medical and Veterinary Sciences, James Cook University, Townsville, QLD 4811, Australia; World Health Organization Collaborating Centre for Vector-Borne and Neglected Tropical Diseases, James Cook University, Townsville, QLD 4811, Australia

**Keywords:** Ashanti region, Ghana, health system, indigenous medicine, integrated healthcare, traditional medicine practitioners

## Abstract

**Background:**

Traditional medicine (TM) plays a vital role in the Ghanaian health system by serving as an alternative healthcare delivery system for the majority of people. However, the quality of practice and level of TM practitioners’ involvement in the integration of TM into the health system have not been fully investigated.

**Methods:**

This study employed a phenomenological qualitative study design to explore the perceptions, experiences and recommendations of TM practitioners in the Ashanti region regarding TM integration. Data were collected through individual interviews with 17 participants.

**Results:**

Participants had knowledge about TM integration. They cited effective alternative healthcare delivery and improved patient outcomes as the key benefits of TM integration. However, they reported a shortage of approved TM products, poor visibility of TM integration and poor relational coordination of care as factors hampering the integration. Participants recommended improved interprofessional relationships, provision of financial support and improved publicity of TM as possible strategies to enhance TM integration in Ghana.

**Conclusions:**

The findings of the study clearly demonstrate that the Ghanaian health system is currently operating a consumer-led, tolerant health system with a parallel (between orthodox and TM practitioners) healthcare delivery model. Successful implementation of an effective TM integration would require improved integrative collaborative coordination of care between orthodox and TM practitioners in Ghana.

## Introduction

Traditional medicine (TM) refers to the sum total of knowledge and practices, whether explicable or not, used in the diagnosis, prevention and elimination of physical, mental and social imbalance, relying exclusively on practical experience and observations handed down from generation to generation, whether verbally or in writing.^1,2^ In Ghana, TM includes the utilisation of plants with medicinal value as well as faith/spiritual healing for therapeutic reasons.^3,4^ TM practitioners are regarded as competent healthcare providers within their communities.^1^ They usually use vegetable, animal and mineral substances for the treatment of illnesses. TM practitioners also employ methods based on the social, cultural and religious background as well as on the knowledge, attitude and beliefs that are prevalent in a given community regarding health, well-being and the causation of disease and disability in the care process.^1,5^ This study focuses on the use of medicinal plants for therapeutic reasons and extends the definition of TM practitioner to include a person who has acquired formal or professional training in the field of TM and has the license to practice.

Studies have highlighted the significant role TM plays in the delivery of healthcare and meeting the health needs of people in both high and low/middle income countries.^6–9^ TM usage has been reported in high-income countries such as France (75%), Australia (48%), Canada (70%), Belgium (38%) and the USA (42%).^9,10^ The WHO has also stated that TM continues to form an integral part of health systems in Latin America, Africa and Asia. For example, in China, TM constitutes approximately 40% of all health services delivered, while traditional forms of Malay, Chinese and Indian medicines are widely used in Malaysia.^10^ Likewise, 80% of the African population relies on TM as the first line of healthcare.^10^ Some Ghanaian studies have stressed that TM serves as the first line of healthcare and is mostly used in the treatment/management of about 42 ailments, particularly cuts, foot rot, stroke, fevers and diabetes.^11,12^

It is reported that TM is easily accessible to Africans.^8,13^ For example, in Uganda, the TM practitioner to clients ratio is 1:200–400 and the orthodox health practitioner to clients ratio is 1:20 000. In Ghana, the ratio of TM practitioners to clients is 1:400 and the orthodox health practitioner to clients ratio is 1:12 000.^3,12^ TM is widely utilised in Ghana. However, the inappropriate use of TM can negatively affect the health of its users. For example, the unregulated use of TM among women of childbearing age in Ghana has led to reproductive health complications such as abortions, ectopic pregnancies and pelvic inflammation diseases among others.^14^ The health complications necessitated the need to regulate TM use and direct service users towards qualified TM practitioners. This in turn facilitated the incorporation of TM into the formal health system in Ghana.

The practice of incorporating TM into formal health systems is becoming an accepted and widely used model in health delivery systems around the globe.^4^ The WHO enlists three forms of TM incorporation, namely, integrative, inclusive and tolerant health systems.^10,12^ These forms of integration differ in terms of the level of inclusion and acceptance of TM integration in formal health systems. For example, a country is said to be practising an integrative health system if TM is formally recognised and integrated in all aspects of healthcare delivery.^8,10,12^ An inclusive health system on the other hand refers to a health system where TM is officially recognised as a medical practice; however, TM practice might not be fully incorporated in all spheres of healthcare, training and education. With tolerant health systems, healthcare is solely based on the orthodox or modern health system, with only certain TM practices being legally accepted.^10,12^ Countries with integrative health systems include China, the Republic of Korea and Vietnam among others. Inclusive health systems are practised in both high (the UK, Germany, Canada and Australia) and low/middle income countries (Ghana, Nigeria, Mali and Equatorial Guinea).^10,12^ The tolerant health system is best exemplified in New Zealand.^15^

In Ghana, the health system is organised in such a way that the orthodox health system is well funded and maintained by policymakers. The traditional health system, on the other hand, although popular, receives less support from policymakers.^16^ The inclusive health system as practised in Ghana means that the country accepts TM as a medical system and has a TM policy, which regulates the practice. The Traditional Medicine Practice Council (TMPC) and Ghana Food and Drug Authority (FDA) were created to license/register TM practitioners and to regulate their activities.^4,17,18^ A Centre for Scientific Research into Plant Medicine has also been instituted to improve TM practice by means of scientifically authenticating the quality and safety of TM products. Other interventions include the creation of the TM department under the Faculty of Pharmacy at the Kwame Nkrumah University of Science and Technology (KNUST), which trains people to become professionals in TM practice and the establishment of TM units in certain public hospitals in Ghana.^4^ These interventions were carried out to increase the credibility of TM in the health system as well as to promote and preserve indigenous medical knowledge.^4,12^

Despite the implementation of these interventions, TM integration in Ghana has not been successful as expected because of weak interprofessional collaboration between TM and orthodox health practitioners^4^ and a low level of awareness concerning the integration process among service users.^16,19^ A study has reported the issue of a ‘power struggle’ between TM and orthodox health practitioners, where TM practitioners want to retain power and control their area of expertise by suggesting co-referral arrangements and local collaborations.^8^ Conversely, orthodox health practitioners prefer the incorporation of TM under the direction of the formal health system, where the TM operates based on the principles of orthodox healthcare.^8^ Clearly, the two groups of health practitioners have different perspectives regarding the implementation of the integration process. To adequately address existing tensions between the two groups and foster better interprofessional collaborations for effective TM integration in Ghana, it is important to explore all key stakeholders’ perceptions of the level and quality of integration of TM into the Ghanaian health system.

**Figure 1.**
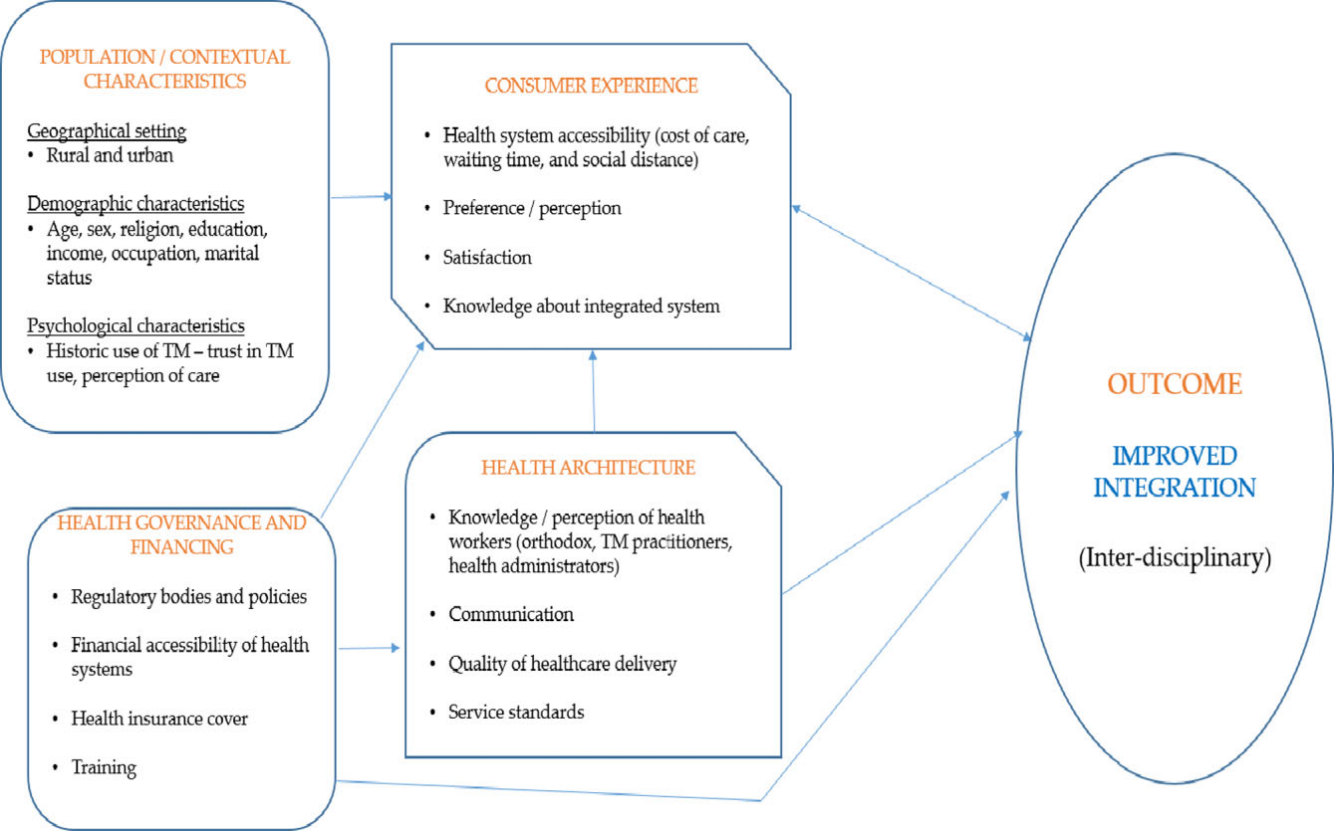
Conceptual framework for integrating TM into health systems. Source: Adapted from Park and Canaway.^15^

The current study is an aspect of a larger research project that used the conceptual framework for integrating TM into national health systems^15^ to assess the practice of TM integration in Ghana. The framework presents four main components that influence TM integration: population characteristics, consumer experiences, health architecture and health governance/financing (Figure 1).

The population characteristics and consumer experiences components of the framework have been employed to evaluate the experiences of health service users,^19^ while the health architecture/health governance and financing components were used to investigate the experiences of orthodox health practitioners and hospital administrators in the Ashanti region of Ghana.^18^ The findings from these studies accentuate that TM integration has not been effective in Ghana. Given that TM practitioners are major stakeholders in the health system, exploring their perceptions and experiences about TM will provide a holistic approach to the practice and integration of TM in Ghana. Therefore, this qualitative study sought to address the following research questions:

- What are the perceptions and experiences of TM practitioners in the Ashanti region regarding the integration of TM practice into the formal Ghanaian health system using a conceptual framework for TM integration?
- What are the recommendations to improve TM integration in Ghana?

The current study was conducted among TM practitioners and it focused on two elements/components of the framework:

1. Health governance and financing.
2. Health architecture.

The use of a conceptual framework in the current study offers adequate insight into the relationship between the key components of TM integration and provides guidance as to how the integration process may be improved in Ghana.

## Material and Methods

### Study design

This study adopted a phenomenological qualitative research design to explore the perceptions, experiences and recommendations of study participants relating to TM integration into the Ghanaian health system. Phenomenology design helps to present the lived experiences of people relating to an event or phenomenon.^20^ Hence, the adoption of phenomenological research design aided in exploring what study participants have experienced, how they acquired the experiences and a collective presentation of such experiences.^20^

### Study setting

The Kumasi metropolis and Offinso north district of the Ashanti region were selected for the study. Details of the study area have been reported in earlier studies.^18,19^ The Ashanti region was selected because it is endowed with a range of TM products as it is located in the forest belt in Ghana.^4^ In addition, the region has a high annual population growth rate of 2.7%.^21^ However, the increase in population size does not tally with the number of health facilities available, causing inadequate health services. Exploring the experiences of TM practitioners to promote effective integration of TM into the orthodox system might expand health services to cater for the growing population in the region. Furthermore, the region is one of the most populous and urbane geographical areas in Ghana with various socioeconomic, ethnic and cultural backgrounds.^4^ Therefore, the multiethnic aspect of the region could enhance the transferability of the study findings.

### Target population and recruitment strategy

The study targeted TM practitioners within the Kumasi metropolis and Offinso north district. These settings were selected with the aim of identifying similar or differing experiences among rural and urban TM practitioners regarding the practice of integration in Ghana. Kumasi metropolis represented the urban setting because it is the regional capital, with the highest population size^22^ and integrated health facilities. Offinso north district was the rural setting because it accounts for the least population^21,23^ with no integrated health facilities.

In this study, a TM practitioner is a person who has practised TM whether in formal (integrated health facilities/TM clinics) or informal (community-based) settings for at least a year in the study settings. Other definite inclusion criteria comprised practitioners who were aged ≥18 y and self-declared knowledge about the use of TM in diagnosing and treating/managing disease conditions. TM practitioners were targeted as stakeholders in the Ghanaian health system given that about 70% of Ghanaians are reported to use TM.^16^ Study participants who practised within formal settings such as integrated health facilities and clinics were recruited purposively, while a snowballing sampling technique was used in recruiting community-based TM practitioners. Thus the recommendations of previously sampled community-based practitioners led to the enlistment of prospective participants.

### Data collection period

Prior to data collection, two research assistants (one male, one female) from the University of Cape Coast were trained in a 5-h workshop on the objective of the study and data collection process, using an interview guide as the training module. The assistants each have a master's degree in Public Health. The training of the research assistants on the study helped them to understand the goal of the research and facilitated consistency in the interview process, as well as the use of the interview guide. Data collection was conducted from mid-March to May 2021.

### Data collection procedure

All interviews were conducted using face-to-face in-depth individual interviews and the languages used were English and Twi (the main local dialect in the study area). English and Twi were used to cater for the varying educational levels of the study participants. The interviews were conducted in conducive environments as chosen by the study participants. All interviews were audio-recorded with the permission of participants and lasted 55–70 min.

Primarily, the practitioners were approached and provided with information sheets, which detailed the objective and benefits of the study, as well as the ethical considerations. Both verbal and written informed consent was obtained from every participant before the start of each interview. It is reported that TM use is predominant in the study setting,^16^ which could give rise to bias in the interview process. To minimise this bias, a training manual was used to educate research assistants as to how the interview questions should be asked. This averted the bestowment of interviewers’ personal preferences concerning TM integration. The first-named author (IGA) was present at the first three interviews to ensure exactitude and uniformity in the interview procedure, but no conversation occurred between the study participants and IGA.

To maintain anonymity, names and other identifiable information were not assigned to the participants; rather, they were allotted numbers. At the onset of the interviews, study participants were required to provide some demographic information (sex, age and years of practice) about themselves. A semistructured interview guide was used that comprised questions relating to the participants’ perceptions, experiences and recommendations as how to improve TM integration in Ghana. Specifically, topics such as perceptions about the health systems, interactions within the health system, regulation of TM practice in Ghana and recommendations to improve the Ghanaian integrated health system were discussed. Data saturation was achieved at the 14th interview, after which three more interviews were conducted, giving a total of 17 interviews. The additional interviews were conducted to ascertain that no extra information collected had distinctive characteristics to form a new group or theme.^24^ The assistants prepared field notes by way of recording their experiences and dealings with the participants. The research assistants are familiar with research methods, particularly in the field of qualitative research. To avoid bias and enhance precision in the data collection, allowable probing questions were included in the interview guide. The inclusion of probing questions prevented the research assistants from introducing their ideas during the interview process. Recurrence of the interview process was not necessary; however, elucidations were acquired from some study participants after the data collection phase.

### Data analysis

An experienced transcriber transcribed all the 17 interviews and the first author (IGA) read the transcripts carefully and thoroughly. NVivo version 12 (QSR International Pty Ltd, Victoria, Australia) was the software used in analysing the data, while framework analysis was the analytical method used. Framework analysis entailed five systematic steps, in which inductive and deductive procedures were employed in the analysis. Then interpretations or synthesis of the findings were performed using the conceptual framework of the study. These five systematic steps include familiarisation; identification of thematic frameworks; indexing; charting; mapping and interpretation.^25,26^

Two authors (IGA and BSMA) conducted data analysis independently. The transcribed data were read in detail on numerous occasions to ensure familiarisation with the data. Then a thematic framework (recognition of main concepts or ideas) was developed based on comments/summaries made at the familiarisation stage. The key ideas narrated by the participants were inductively ascertained at the thematic framework stage. Indexing was performed by placing parts of the data under specific broad concepts or themes. An inductive method of analysis was also applied at this stage, ensuring that the themes were generated from the data. Then charting was conducted by organising the marked data in charts according to the themes identified. Lastly, mapping and interpretation were carried out by arranging the charted data to demonstrate the study participants’ perceptions and experiences with TM integration into the Ghanaian health system. During the mapping and interpretation stage, a deductive analytical method was applied in categorising the themes under the elements of the utilised conceptual framework.

To ensure further rigour in the data analysis, initiatory coding and generation of themes were performed separately by IGA and BSMA. Discrepancies between the coders were resolved through discussions at a consensus meeting. The other authors (AAS, AEOMA and TIE) reviewed the themes and quotations to enhance the trustworthiness of the study findings. The elements of trustworthiness (credibility, dependability and transferability) were ensured through investigator triangulation, supervisor/peer debriefing, member checking and detailed description of the study site and methods used. The themes are presented with demonstrative quotations followed by each subject’s details (e.g., ‘Participant 1, Kumasi’). The Consolidated Criteria for Reporting Qualitative Studies (COREQ) checklist^27^ for reporting qualitative studies was used in reporting the study (see Supplementary File 1, COREQ Checklist).

## Results

### Characteristics of study participants

In the Kumasi metropolis, there are nine TM practitioners practising in integrated health facilities. Six out of the nine practitioners agreed to participate in the study. Eleven participants were purposively sampled from non-integrated health facilities, totalling 17 interviewees. Of the 17 participants, 10 practised in the Kumasi metropolis, whereas the remaining were located in Offinso north district. Most (six) of the participants from the Offinso north district were community-based practitioners. The majority (11) of the participants were males. Participants were aged 26–71 y. Seven participants had a tertiary level of education. On average, participants had about 13 y of practice experience.

### Themes

In all, eight themes emerged from the analysis of participants’ responses. Three themes (efficacy of traditional health therapies, a patient-centred approach to TM delivery and effective alternative healthcare delivery) enabled the integration process. While the remaining five themes (financial constraints associated with TM practice processes, poor quality of TM operational processes, unbalanced professional training opportunities, poor relational coordination of care and poor visibility of TM integration) were identified as barriers to TM integration in Ghana.

#### Enablers of TM integration

##### Efficacy of traditional health therapies

Study participants started by expressing their positive notions about traditional health therapies. They expressed the view that traditional health therapies, particularly TM products, were associated with negligible undesirable effects. The rationale behind participants’ assessment was that orthodox medicines have been processed, therefore contain some form of chemicals, but TM is natural and hence has little or no inimical effect. Participants, regardless of the location of operation (whether solely or within an integrated facility), believed that TM is effective in treating maladies, particularly fevers.

*…[A] client was taking orthodox medicines for typhoid but he was not getting better. So, it was a worry as he kept complaining about abdominal pain and growing lean. So, I asked the client to go for a lab test, which showed that he had typhoid fever. So, I administered the TM to him and after taking two or three courses of the TM, he saw massive improvements* (Participant 5, Kumasi).

*The orthodox medicine has been synthesised but the TM is natural so it is associated with little or no side effects* (Participant 12, Offinso north).

##### Patient-centred approach to TM delivery

When discussing TM practitioners’ approach to healthcare delivery, the participants emphasised the importance of a patient-centred approach to healthcare delivery, which they employed in the care process. They maintained that they listened effectively to their clients’ concerns and showed genuine care towards them. They believed that the patient-centred approach made the clients feel welcomed and appreciated, which fostered improved patient health outcomes. In addition, the majority of the participants emphasised that service users demonstrated their satisfaction with the services they received through recommendations. According to them, once a service user was satisfied, they recommended the TM practitioner's services to other people/potential clients or showed appreciation by thanking them in person.

*When they [clients] come, I take time to listen to their concerns; I make them feel welcomed and accepted. Therefore, they get the assurance that I will provide them with the treatment and I always do* (Participant 7, Offinso north).

*When they come here and they are satisfied, they recommend our services to more people. Sometimes, some of them would come and thank you or show appreciation* (Participant 15, Offinso north).

##### Effective alternative healthcare delivery

Formal training on TM bridges the gap. TM practitioners operating within integrated health facilities have formal training; therefore, they adopt a scientific approach in diagnosing disease conditions and their healing processes are in line with acceptable scientific procedures in healthcare, which foster effective and safe alternative healthcare delivery. These practitioners perceive that the integration intervention bridges the gap between orthodox and indigenous means of healthcare delivery. TM practitioners operating in integrated health facilities identified the availability of different healthcare services as an advantage of incorporating TM into the Ghanaian formal health system. They insisted that TM integration has provided effective options in healthcare services, whereby service users alternate between the two health systems until they achieve the expected outcome.

*Now, there is an alternative, people can choose which healthcare system they want. Also, where they [clients] do not see improvement in accessing orthodox healthcare, they come to TM and it works* (Participant 4, Kumasi).

*…[T]he beauty about the current state we have is that we have a professional who lies in between the orthodox setting and the traditional setting that is the medical herbalist who is trained to understand orthodox medicine or style of healthcare and also trained to understand TM. So that, the trained TM practitioners’ approach in diagnosing disease conditions in the health team follows the scientific processes* (Participant 16, Kumasi).

#### Barriers to TM integration

##### Financial constraints associated with TM practice processes

The participants demonstrated good knowledge and understanding of the role and responsibilities of the regulatory bodies and the relevant policies. They also reported familiarity with the FDA, the TMPC and the TM Act that regulate TM practice in Ghana. They mentioned that the FDA is responsible for the training of TM practitioners and the issuing of licenses after evaluation of TM products. According to the participants, the TM Act was passed to regulate the entire TM practice, while the TMPC registers practitioners as well as their places of operation.

*The TM council with the Acts, Acts 575 and FDA regulate TM. It regulates the practitioners. In the first place, it registers TM practitioners and their premises. So, if this is the premises that the practitioner practices, then the TM practitioner would have to first register his/herself, and then you register the premises* (Participant 2, Kumasi).

Study participants were satisfied with the operations of the supervisory bodies; yet they felt that the cost of their services was too expensive. They believed that the FDA does not consider the economic predicament of some of the TM practitioners and based this conclusion on the exorbitant charges of the FDA. They explained that the very expensive charges of the FDA serve as an obstacle to proper TM practice in Ghana (an inability to produce standardised products/services).

*The FDA performs considerably well just that their price is too expensive. They do not consider the economic predicament of some of the practitioners. If you do not have money, then you cannot offer standard services or products. Hence, not practising proper TM* (Participant 12, Offinso north).

Furthermore, the participants identified a number of financial bottlenecks that affect the proper execution of TM practice in Ghana. These financial bottlenecks include the high cost of TM practice registration, partial coverage of TM in the National Health Insurance Scheme (NHIS) and the exorbitant cost of approved TM products. Primarily, participants described the TM registration process as burdensome. They based their evaluation mainly on the cost involved in setting up the required physical structure for the processing of the products, as well as the cost of having products tested for safety at approved scientific research centres or pharmacological units. Participants from the rural setting clearly stated that they do not earn much from the practice; therefore, making certain payments to register their practice/products is not an easy task for them.

*The regulatory processes are not easy at all, even in terms of money. The facilities that should be registered for TM practice should have a minimum of four bedrooms. You know how difficult it is to get a one-bedroom structure in Ghana, not to think about four and these four rooms are not meant for sleeping; this is where you will prepare the TM. So, you can imagine! Getting water to your facility should be something that will not rust. So, water alone will cost you Ghana Cedis [GHC]15 000. So, getting the facility ready costs billions…before you can produce any TM product, they will come and inspect your facility. You cannot say that you going to sit in your house and do anything. They will scrutinise everything. In addition, they will let you bring the products to the pharmacological unit for further analysis at KNUST. It is based on the results that they [the FDA] will issue you a license* (Participant 6, Kumasi).

*…[W]hen I was going through the process, they asked me questions about the ingredients I used in preparing my medicine. They asked me to take the medicine to a Centre for Scientific Research where they run some test on the medicine. After that, you will have to make some payments. After the test in the city, you will have to make payment and many bureaucracies that you have to go through. I do not remember the exact amount that you have to pay but I spend over GHC4000 on the processes. We do not make that kind of money so when we were supposed to go and make that payment, it was not easy at all* (Participant 7, Offinso north).

The narratives from the participants in integrated health facilities depicted that the traditional health system is partially covered by the Ghanaian health insurance scheme and it is limited to the government facilities selected to deliver integrated healthcare. Participants from non-integrated health facilities also confirmed this notion; they stated that clients pay for consultation and TM products because their services and products are not included in the national health insurance scheme. Study participants believed that the exclusion of TM products from the health insurance scheme accounts for the low patronage of healthcare services at integrated health facilities.

*…[W]ith the orthodox, both the service and medications are covered by the health insurance. But for TM section, the service is covered by the health insurance but the products are not. The medicine itself is not covered by the health insurance and so patients are supposed to pay for those TM products. So, if in the same hospital, you will not pay anything for a particular type of treatment but for the other you will pay, then definitely, you will go for the one that you will not have to pay. It is obvious. It is not helpful because we do not get many clients* (Participant 5, Kumasi).

*I have come across some clients who were really sick and did not have money yet had the NHIS cards but I cannot accept the cards because my services are not included in the insurance scheme. When I accept the NHIS cards, I will run a loss, which is bad for business. So clients have to pay for both the services and the products* (Participant 14, Offinso north).

TM practitioners who operated in the integrated health facilities further explained that exorbitant costs of approved TM products were a contributory factor to the ineffective nature of TM integration in Ghana. They felt that the cost of treating ailments with approved TM products was higher than orthodox medical treatments. They clarified that healthcare services delivered in such facilities tend to be more economical than in private TM clinics because service users do not pay consultation fees.

*For antimalarial, when you come to the TM side, we will give you three bottles that you have to take for seven days. That can cost you GHC60 but when you go to the orthodox side, the antimalarial is for three days and you will spend about GHC20 on it. I think the difference is too much* (Participant 5, Kumasi).

*When they [clients] come here [the TM unit], the cost of care is less expensive than going to a private facility because consultation is free* (Participant 4, Kumasi).

##### Poor quality of TM operational processes

Another barrier to TM integration is the poor quality of TM operational processes. Participants, irrespective of the location of operation, identified standard/good TM operational processes (proper certification, packaging, storage of TM, products and constant supply of approved TM) as a fundamental strategy required to standardise TM practice and promote its integration. However, some of the study participants admitted that they do not package their products to meet acceptable standards, whereas others confessed that they fail to provide necessary information about their products. Participants associated their failure to embark on proper packaging and certification of products to cost. The issue of poor certification was prevalent among participants in the rural setting.

*With the packaging, it is good but I do not do it. Packaging, labelling and all those things come with huge cost but I do not earn much from the practice so I am unable to package nicely* (Participant 12, Offinso north).

In addition, participants expressed their displeasure with the ways in which TM products are displayed and stored. They felt that some practitioners do not adhere to proper storage standards, especially during sales, hence they believed that poor storage could lead to harmful storage reaction conditions, where users would not achieve the best results from using the products.

*Storage of TM is an issue. Even in the chemical shops where the medicines are displayed for sales, very few of them have air conditions in the facilities. We know that drugs are to be stored in cool places and far away from direct contact from sunlight. Some of these medicines are not packed in sunbath bottles and all that. Of course! We are subjecting the product to harmful or other storage reaction conditions, which is an issue. When these reactions take place, people will not get the best out of the products* (Participant 16, Kumasi).

Regarding the supply of TM products in the integrated health system, TM practitioners within integrated health facilities mentioned that countless TM products are available in the Ghanaian health system; however, only products that are properly certified, approved by the Ministry of Health and included on the list of essential drugs are supplied to the integrated health facilities. They perceived that although there are variations in the supply of orthodox medicines, the same could not be said of the approved TM products. The participants recounted that they usually experience a shortage of the products due to the restrictive nature of the supply. They attributed the shortages of approved TM in the integrated facilities to the extensive procurement system in the government sector. Participants concluded that the shortage of approved TM products interferes with service delivery and subjects the service users to unnecessary delay in receiving healthcare, which in some instances has led to the loss of clients.

*Shortage of the TM products is one of the problems because the drugs are being provided by the Ministry of Health. There are always shortages of these drugs because there are no varieties as compared to the orthodox medicines. The drugs are limited but we have the traditional people who can get the drugs but they are not certified by the Ministry, therefore, we cannot use it in the hospital. For example, Taabea TM products are very good but it cannot be used in the hospital because it is not certified as been part of the essential list* (Participant 17, Kumasi).

*Currently, when the TM products get finished, you have to wait for about three or four months before getting stocked. By then, you may lose some of your clients* (Participant 5, Kumasi).

##### Unbalanced professional training opportunities

The interviews indicated that some of the participants were not impressed with the level of professional training in their practice. They felt that the acquisition of formal training among TM practitioners was low because some of their colleagues gained knowledge on practice in an unofficial manner, in particular apprenticeships through family lineage. They explained that the informal nature of acquisition of knowledge regarding the practice means that some practitioners lack the basic knowledge of standard medical practice. When asked to disclose the forms of training they had received, the narratives of those from an urban setting showed that they received training on TM practice through formal education, whereas rural practitioners learnt the profession informally by way of family heritage.

*Most of the TM practitioners have not had formal education. They learnt from their fathers. They may have some knowledge on medicinal plants and treatment of common illnesses but that is not enough because they will be lacking some basic skills or standards in medical practice* (Participant 11, Offinso north).

*I had my training at KNUST and Mampong. At KNUST, I spend four years before moving to Mampong for an additional two years of training. So, in all, I spent six years of training on TM* (Participant 5, Kumasi).

The participants who acquired formal training on TM at the KNUST described the nature of the training they received. They described themselves as clinicians because they were exposed to courses such as biochemistry, anatomy, physiology, clinical pathology, general pharmacology dispensary and TM. They believed that undertaking such courses helped them acquire both orthodox and traditional medical knowledge.

*I was trained as a clinician; I was also trained in the area of diagnostic health that is clinical diagnostic, general pharmacology and pharmaceutical chemistry. The courses that are related to pharmacy. I also did clinical pathology, biochemistry, anatomy, physiology, TM, and a little bit of social functioning and English* (Participant 16, Kumasi).

*At our faculty, we do pharmacology; the study of plants from first to final year. We also do formal medical courses as well, such as anatomy, physiology, pathology, biochemistry. We also do an aspect of pharmaceutical courses mainly dispensary. We also go for clinical…we do it from first to final year. Finally, we do some courses at the medical school and pharmacy courses as well* (Participant 17, Kumasi).

##### Poor relational coordination of care

The participants who practised in integrated health facilities reported that orthodox health practitioners oppose TM integration by urging service users not to seek care at the TM units. Some participants stressed that orthodox health practitioners, particularly medical doctors and nurses, resist TM usage because they believe that successful TM integration into the Ghanaian health system would decrease the customer base of the orthodox health system, thereby increasing that of TM practitioners.

*Some of the nurses even give the patients directions to go to different places for treatment and those patients who really know about the TM will still insist on coming to the TM unit for treatment. Some of the nurses and medical doctors even sack the patients. It got to a time we had to report the issue to their boss at the hospital to caution the nurses. The behaviours of these orthodox health professionals deter the patients from seeking TM at the hospital* (Participant 17, Kumasi).

*Some people think that when TM is successfully integrated, we [TM practitioners] would be taking their jobs. That is what some of the orthodox medical practitioners think, particularly the medical doctors so they resist TM and do not refer patients to us* (Participant 15, Offinso north).

Study participants indicated that active interaction between TM and orthodox health practitioners has the ability to expedite TM integration into the formal health system. However, they viewed the nature of referral within the Ghanaian health system to be intrareferral (referral among TM practitioners) rather than cross-referral. They explained that intrareferrals were carried out because of the absence of specific TM products at some integrated health facilities and inadequate technical knowledge to treat a particular disease condition.

*Some of our colleague TM practitioners do refer patients to us. A colleague in Accra may even ask someone to come and see us. Sometimes, if the facilities do not have a particular TM medication for patients, they refer them to come here. For example, there was a woman who was receiving treatment for fertility issues. When she came to our facility, we did not have the medication for her so we referred her to Atonsu Agogo [Kumasi South Hospital]. Now, when Atonsu Agogo Hospital has a problem, they also refer their TM patients to us* (Participant 5, Kumasi).

*No single person can treat all kinds of diseases. Everyone specialises in a particular area. So, if a patient come to me with a disease that I cannot treat, I refer him/her to a colleague who treats that disease. Personally, I have not had any referral from the medical doctors. Rather, I have received some referrals from my fellow TM practitioners* (Participant 9, Kumasi).

##### Poor visibility of TM integration

Another major barrier highlighted by the participants is the poor visibility of TM integration. Most participants in the urban setting felt publicity about integrated health facilities was not enough; hence, the value of their practice is not evident. They explained that publicising TM integration is supposed to be a nationwide exercise, spearheaded by the government (officials in charge). Nonetheless, they felt that publicity has been relegated to the health facilities. Participants considered inadequate publicity about TM integration as the cause of low awareness levels among service users in the Ashanti region.

*They [the officials in charge] have not done so well with publicity. We tell people about the service so that they come. It is supposed to be a national thing but they are not doing it* (Participant 2, Kumasi).

*There are a lot of people who do not know about the existence of TM in the government hospitals due to low publicity about the integration* (Participant 4, Kumasi).

##### Participants’ recommendations to foster better integration of TM into the health system

**Figure 2.**
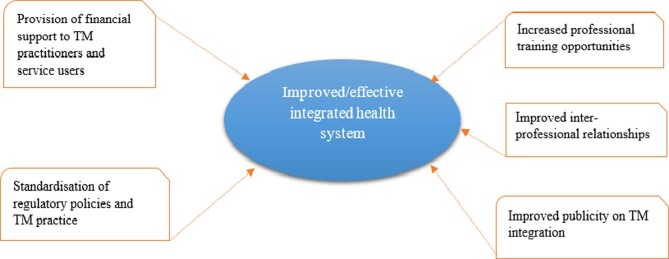
Study participants’ recommendations to foster better integration of TM into the Ghanaian health system.

In the light of all the barriers impeding the integration process, the participants believed that TM could properly be integrated into the Ghanaian health system. On that note, they proffered five major recommendations to policymakers, namely, the provision of financial support to TM practitioners and service users, standardisation of regulatory policies and TM practice, increased professional training opportunities, improved interprofessional relationships and improved publicity of TM integration (Figure 2).

##### Provision of financial support to TM practitioners and service users

From a financial support point of view, the participants suggested that to improve TM integration would require the provision of financial support by the government to TM practitioners. They anticipated that community-based TM practitioners’ ability to access loans or financial support would help to upgrade their activities by adopting proper manufacturing and packaging procedures. The participants believed that the adoption of proper manufacturing and packaging processes would help standardise their products and subsequently advance TM practice.

*Everything we do revolves around money. So, we need the government to provide us with financial resources. With the financial support, we can embark on proper manufacturing and packaging processes and it will elevate the standard of TM practice* (Participant 14, Offinso north).

To financially support service users, the majority of the study participants proposed that TM products should be included in the NHIS. They indicated that most rural residents depend solely on farming as their source of income. Therefore, they live in poverty during out-of-season periods. The participants believed that the inclusion of TM products in the NHIS could serve as a motivation for service users, particularly the poor, to seek care at approved TM health facilities when the need arises, which will consequently improve the health of rural residents.

*If TM is included in the national health insurance, it would be great. Because when you come to our villages, most of the people are farmers. In Offinso north, the people are into cocoa farming. So, there are specific times that there is money in the community. That is, during the harvesting season. After that period, you have to live in poverty until the next season. So, if someone gets sick out of season, it becomes difficult. If TM is included in the NHIS, it will help support the health of the people because TM is the common healthcare here* (Participant 7, Offinso north).

### Standardisation of regulatory policies and TM practice

Although participants were pleased with the performance of regulatory bodies, they recommended that for TM to be properly integrated into the mainstream health system, the authorised agencies would need to implement strict rules and regulations in governing TM practice. They envisaged that the creation of government-owned manufacturing sites in the various districts could aid the effective implementation of TM regulatory measures in the area of supervision, monitoring and evaluation.

*The government should build manufacturing premises for the districts. So, the premise will be for the government, and they will be able to effectively implement regulatory rules, be it supervision, monitoring and evaluation* (Participant 11, Offinso north).

### Increased professional training opportunities

Clearly, some of the participants had a strong conviction that formal training of TM practitioners has a crucial role to play in delivering the best healthcare to service users and promoting TM integration. Due to this belief, they indicated that TM training institutions should be increased to widen the scope of TM practice in Ghana. They indicated that the expansion of TM training institutions would grant people who are interested in the field, the opportunity to develop their skills and acquire knowledge of the practice through formal education. Participants were convinced that widening the scope of the practice would increase the admiration of people for that field of medicine. This narrative was highly endorsed by the urban participants. Similarly, the rural participants felt that offering professional training to TM practitioners could improve integration because the country would have a pool of professional TM practitioners to complement the orthodox healthcare system.

*All health institutions should run a little bit of courses on TM. That way, it will widen the scope of TM training in Ghana. It will help people to appreciate the practice* (Participant 3, Kumasi).

*The government should increase the schools that provide training on TM. Currently, aside KNUST, there is no other government institution that provides TM training. So, I recommend that all the universities in Ghana should mount programmes in TM so that those who are interested in it can go there and develop their knowledge and skills* (Participant 1, Kumasi).

*The government can arrange to train TM practitioners who are already in the field so that at the end, we will have a pool of professional TM practitioners that they can choose from to complement the orthodox health system* (Participant 15, Offinso north).

### Improved interprofessional relationships

To address the issue of the orthodox health practitioners opposing TM usage, the participants recommended that the leaders of the TM practitioners’ Council should coordinate and liaise with the Ghana Medical Council to accept the incorporation of their practice into the formal health system. They deemed the Medical Council as vocal and influential in matters relating to national health policies; therefore, they believed that the council's support would boost the government's commitment towards the integration process.

*The Medical Association is very vocal and are very influential when it comes to national policies. So, our leaders [TM association, TM council] should also push things and liaise with the Medical council so that the Council can accept the integration. Without the approval of the Medical Council, there is no way the government will listen to us and promote the integration* (Participant 9, Kumasi).

### Improved publicity of TM integration

All participants noted that one key issue that needs to be addressed to enhance TM integration is sensitising the public on the integration process, particularly on the existence of TM units in some government hospitals using the media as a means of communication.

*We have to educate people that when they go to the hospital, there are orthodox medicines and TM. The issue is that, for many people, when they go to the hospital, they do not expect to receive TM but rather orthodox medicine. So, if we create awareness through posters, radio and television, people will become conscious that when they go to the hospital, they can have access to both orthodox and TM* (Participant 8, Kumasi).

*….[S]ome TM practitioners are well known. They have access to the media such as the TV and radio stations. So, we the less known TM practitioners can collaborate with them to promote TM practice and its integration using the various media platforms. That way, people will know about the integration process as well as where to access integrated healthcare or approved TM services/products* (Participant 10, Offinso north).

## Discussion

The integration of TM into the Ghanaian health system has been an issue of interest for many stakeholders, including service users, orthodox medicine and TM practitioners. The current study explored the perceptions, experiences and recommendations of TM practitioners in the Ashanti region pertaining to the integration of TM into the Ghanaian health system using a conceptual framework for integrating TM with national health systems.^15^ The study was founded on two constituents of the framework: health governance and financing and health architecture. The participants’ recommendations for better TM integration in Ghana are one of the study's major additions to the body of knowledge on health systems integration.

Previous studies have recounted that TM use is predominant in Africa and Asia.^28^ Researchers have cited factors such as TM aligning with cultural beliefs, effectiveness and minimal side effects as reasons for its use.^19,29^ Such findings have been substantiated in the current study, where participants mentioned that TM products are effective in treating illnesses such as fevers with minimal adverse effects. The effectiveness of TM as recounted by healthcare practitioners^18,29^ could be a facilitating factor in the Ghanaian integration process.

TM practitioners in this study narrated that they are more accommodating in delivering integrated care than orthodox health practitioners. Participants indicated that they adopt a more patient-centred or psychosomatic approach to healing, where they focus on discussing psychosocial issues rather than focusing only on medical questions. A similar finding has been documented in a consumer-based study in the Ashanti region where service users attested to the fact that TM practitioners adopt a more compassionate approach to healthcare delivery than orthodox health practitioners do.^19^ This implies that participants approach their clients by exploring their beliefs and fears, which they believe boosts the healthcare process. This attitude of TM practitioners might motivate service users to continue to patronise TM healthcare services irrespective of the cost of treatment and mode of delivery. The government, health practitioners and hospital administrators should endeavour to emphasise a more patient-centred Ghanaian healthcare system.

A systematic review of the practice of integrated healthcare in Africa has reported that TM integration in most African countries is not effective.^30^ Similarly, orthodox health practitioners and hospital administrators in the Ashanti region have identified expensive TM products, poor publicity of integration and poor service standards in the TM field as issues obstructing TM integration in Ghana.^18^ These findings are also confirmed in the current study.

The health governance and financing set-up in Ghana appear to be inadequate to achieve successful TM integration because they contribute to major setbacks in the integration process. These setbacks revolve around financial constraints associated with TM regulatory practice processes and lopsided TM training opportunities for the practitioners. Although the government recognises the significant role TM plays in the Ghanaian health system and has therefore initiated its integration through the creation of the TMPC, the TM Act and the FDA to regulate TM practice,^4,25,31^ a previous study reported that orthodox health practitioners and hospital administrators in the Ashanti region perceived the operations of TM regulatory bodies to be appalling because of the unreliable regulation of TM practice in Ghana.^18^ In the current study, the participants, even although pleased with the services of the regulatory bodies, indicated that the high service fees charged by the regulatory bodies serve as an obstruction to good TM practice in Ghana. The participants stated that most rural TM practitioners do not adhere to standardised medical practice (processing, certification protocols, licensing) due to financial constraints, yet they operate within the communities. The inability of such practitioners to register their products/services exposes clients to unapproved TM products/services, which could be detrimental to their health. This finding clearly indicates that financial constraints as experienced by some TM practitioners in Ghana in getting their practice registered is a major impediment to successful TM integration.

Additionally, the traditional health system does not receive adequate support from the central government, particularly in the areas of infrastructure and financing (NHIS coverage). This finding corroborates earlier studies conducted among health systems researchers,^25^ service users^19^ and orthodox health practitioners/health administrators.^18^ This indicates a sustainable financing scheme would be required to support TM practice in Ghana. One possible strategy would be to incorporate the traditional health system into the NHIS. For example, Switzerland's compulsory health insurance scheme covers certain complementary therapies if the practitioner is trained and licensed to practice complementary medicine.^25,32^ This approach could be adopted in Ghana.

Furthermore, some of the participants expressed a desire for all TM practitioners to be professionally trained and/or acquire formal training in TM practice. This desire has also been established among orthodox health practitioners in the Greater Accra and Ashanti regions of Ghana.^18,33^ The current study has shown that TM practitioners who operate in urban areas tend to have more formal training compared with their rural counterparts and are therefore in a better position to provide appropriate medical advice on the use, misuse, abuse and possible negative effects of TM products. Inadequate formal education or professional training among TM practitioners, particularly those in rural areas, has labelled them as ignorant and less competent than their orthodox counterparts.^8^ The imbalance in formal training leads to relational power disparity and ineffective collaboration between TM and orthodox health practitioners in Ghana and beyond.^4,8,34–38^ For example, a Tanzanian study has reported that orthodox health practitioners do not refer service users to TM practitioners because they perceive them to be uneducated and ignorant.^35^ Similarly, Ampomah et al.^18^ have also disclosed a weak and casual nature of cross-referrals between TM and orthodox health practitioners, particularly in the Ashanti region of Ghana.^18^ Formal training for TM practitioners and quality assured regulatory processes could boost orthodox health practitioners’ confidence in TM practice and improve the interprofessional referral process. Based on these findings, there is a clear indication that efforts to achieve successful integration partly rely on proper TM education, financing and implementation of TM regulatory rules by designated bodies to guarantee the safety and efficacy of TM products in Ghana.

Relating to the Ghanaian health architecture, the study contends that, to promote TM integration in Ghana would require improved interprofessional collaboration between TM and orthodox health practitioners as well as good relational coordination of care. Improved interprofessional collaboration could focus on the establishment of an effective cross-referral system. Referral of service users between healthcare practitioners has been reported in the literature.^4,34,39,40^ The nature of referral in the Ghanaian health system was described as an intrareferral system, where most of the referrals occurred among only the TM practitioners. This indicates that cross-referrals were minimal in the health system, a finding already established by Ampomah et al.^18^ A properly organised referral system might be an efficient strategy to integrate TM into the Ghanaian health system. Therefore, policymakers should take into account the inclusion of a cross-referral model in Ghana's health policies and guidelines.

Even although cross-referral was reported to be weak in the health system, most of the participants demonstrated a strong inclination towards the practice of TM integration in Ghana. The availability of an effective alternative healthcare treatment option within the integrated system explains why, even in the absence of a proper referral system and opposition from orthodox health practitioners, participants still support the incorporation of their practice into the mainstream health system. This benefit of TM integration has also been proven in an earlier study.^18^ Clearly, positive outcomes of integration are serving as a strong motivation for TM integration in Ghana.

The literature indicates that Ghana is practising an inclusive health system.^10,12^ However, the findings of the study report otherwise. This study has highlighted some challenges hindering TM integration in Ghana. These challenges include financial constraints associated with TM regulatory practice processes, poor quality of TM operational processes including shortage of approved TM products, unbalanced TM training and poor relational coordination of care. Concerns over the poor quality of operational processes in the TM field have been recounted in previous studies.^2,33,40,41^ TM products are deemed unsafe and inferior due to the absence of detailed and vital information such as expiration dates, dosages and the conditions under which the products/medicines are processed.^18,33^ This negative notion discourages most orthodox health practitioners from recommending TM products to service users.^18^ These issues prove that Ghana is operating a consumer-led tolerant health system with a parallel health delivery model. Public awareness and health education programmes that aid dissemination of information among health practitioners and professional training of TM practitioners could enlighten and facilitate appropriate/favourable perceptions about TM and its integration into the mainstream health system.^8,31^ Participants also emphasised the need for the provision of financial support to TM practitioners and improved interprofessional collaboration facilitated by the government and leaders of the TMPC as additional ways to enhance TM integration in Ghana.

### Implication for practice

The health goal Sustainable Development Goal 3 (SDG 3) strives to safeguard health and well-being for all individuals at every stage of life.^42^ SDG 3 is established on nine targets including the provision of universal health coverage and enhancing the healthcare industry.^43^ Target 3.8 aims at achieving universal health coverage, including access to safe, effective, quality and affordable medicines for all.^44^ The practice of integrating TM in Ghana is a way of directing the populace towards trained TM practitioners who apply scientific principles and standardisation in their operations.^16^ Such practitioners usually operate in urban areas, as depicted in this study. The availability of trained TM practitioners/approved products, improved TM practice in Ghana,^19^ prevalent TM use,^45^ coupled with its reported effectiveness, could widen the range of healthcare services in the country, and serve as a positive step towards the achievement of universal health coverage. Nonetheless, unbalanced professional training between urban and rural TM practitioners in Ghana could be a major factor impeding the integration process. The activities of untrained TM practitioners in rural areas might continue to hinder the integration process because they may cause more harm than good in the health system, thereby reversing the positive impact urban TM practitioners might have made.

Achieving the health goal in Ghana could also be delayed by the poor quality of TM operational processes/shortage of approved TM products, the high cost of such products and poor quality of relational coordination of care, which serve as barriers to effective TM integration. Strategies needed to enhance TM integration and attainment of SDG 3 include active involvement of the Ghanaian government and policymakers in the following:

- Provision of a comprehensive health financing policy that includes both orthodox medicine and TM products.
- Investment in the health workforce through education to expose both groups of health practitioners to the basic ideologies of the two health systems. This strategy might improve the level of formal or professional training among TM practitioners, thereby fostering better integration and achievement of the SDG 3 health goal.
- Promotion of medical research and investment in research infrastructure, particularly in the field of TM.

### Strengths and limitations

A key strength of the current study is the use of a qualitative research approach to explore participants’ perceptions, experiences and recommendations for improved TM integration in Ghana. This study contributes to the literature in the field of health systems research by highlighting the benefits of TM integration in Ghana as well as elucidating the bottlenecks in the Ghanaian health architecture and health governance/financing structures regarding the TM integration process. The study proffered possible pragmatic solutions to improve TM integration into the Ghanaian health system. The findings of this study may be useful in guiding policymakers in the review of current health policies and guidelines to enhance TM integration in Ghana. Additionally, the inclusion of participants from both rural and urban settings (Kumasi metropolis and Offinso north district) increases the transferability of the study findings. The major limitations of this study include selection bias, that is, the participants may have been people who are interested in the concept of TM integration. Also, the perceptions of policymakers such as the leaders of the TMPC could have been explored.

## Conclusions

This study explored the perceptions, experiences and recommendations of TM practitioners regarding the integration of their practice into the Ghanaian formal health system. The findings of the study show that the practice of TM integration is highly acceptable among TM practitioners in the Ashanti region. However, issues such as financial constraint, unbalanced professional training opportunities, poor TM practices and poor relational coordination of care between orthodox health practitioners and TM practitioners were identified as bottlenecks that hampered the TM integration process. These findings clearly indicate that the Ghanaian health system is currently operating a parallel health delivery model—in which both types of health practitioner independently deliver services within their officially defined domain of practice—rather than one where coordinated integrated interactions take place among stakeholders in the health system. Movement towards an effective integrated health system in Ghana would require better interprofessional collaboration between orthodox and TM practitioners. Future research could focus on intervention studies that address methods by which advanced integrative practice and improved interprofessional cooperation between the two health systems could be developed in Ghana.

## Supplementary Material

ihac059_Supplemental_File

## Data Availability

The data reported in this study/research are available on request from authors.
