# Supplementary material for: Integrating traditional medicine into the Ghanaian health system: perceptions and experiences of traditional medicine practitioners in the Ashanti region
Source: Int Health. 2022 Sep 5;15(4):414–27. doi: 10.1093/inthealth/ihac059 (PMC10318977; doi:10.1093/inthealth/ihac059)
Supplement: ihac059_Supplemental_File [file ihac059_supplemental_file.pdf]

Consolidated criteria for reporting qualitative studies (COREQ): 32-item checklist

No. Item	Guide questions/description	Reported on Page #
**Domain 1: Research team and reflexivity**		
Personal Characteristics		
1. Inter viewer/facilitator	Which author/s conducted the inter view or focus group?	Methods, p 4
2. Credentials	What were the researcher's credentials?	Methods, p 4
3. Occupation	What was the occupation of the researchers at the time of the study? Academics/lecturer	P 1
4. Gender	Was the researcher male or female?	Methods, p 4
5. Experience and training	What experience or training did the researcher have?	Methods, p 4
Relationship with participants		
6. Relationship established	Was a relationship established prior to study commencement?	Methods, p 4
7. Participant knowledge of the interviewer	What did the participants know about the researcher? e.g. personal goals, reasons for doing the research	N/A
8. Interviewer characteristics	What characteristics were reported about the inter viewer/facilitator? e.g. Bias, assumptions, reasons and interests in the research topic	N/A
**Domain 2: study design**		
Theoretical framework		
9. Methodological orientation and Theory	What methodological orientation was stated to underpin the study? e.g. grounded theory, discourse analysis, ethnography, phenomenology, content analysis	Methods, p 3
Participant selection		
10. Sampling	How were participants selected? e.g. purposive, convenience, consecutive, snowball	Methods, p 4
11. Method of approach	How were participants approached? e.g. face-to-face, telephone, mail, email	Methods, p 4
12. Sample size	How many participants were in the study?	Methods, p 4
13. Non-participation	How many people refused to participate or dropped out? Reasons?	N/A
Setting		
14. Setting of data collection	Where was the data collected? e.g. home, clinic, workplace	Methods, p 4
15. Presence of non-participants	Was anyone else present besides the participants and researchers?	Methods, p 4

16. Description of sample	What are the important characteristics of the sample? e.g. demographic data, date	Results, p 5
Data collection		
17. Interview guide	Were questions, prompts, guides provided by the authors? Was it pilot tested?	Methods, p 4
18. Repeat interviews	Were repeat interviews carried out? No	Methods, p 4
19. Audio/visual recording	Did the research use audio or visual recording to collect the data?	Methods, p 4
20. Field notes	Were field notes made during and/or after the interview or focus group?	Methods, p 4
21. Duration	What was the duration of the interviews or focus group?	Methods, p 4
22. Data saturation	Was data saturation discussed?	Methods, p 4
23. Transcripts returned	Were transcripts returned to participants for comment and/or correction?	N/A
**Domain 3: analysis and findings**		
Data analysis		
24. Number of data coders	How many data coders coded the data?	Methods, p 4
25. Description of the coding tree	Did authors provide a description of the coding tree?	N/A
26. Derivation of themes	Were themes identified in advance or derived from the data? No	Methods, p 4
27. Software	What software, if applicable, was used to manage the data?	NVivo, p 4
28. Participant checking	Did participants provide feedback on the findings?	N/A
Reporting		
29. Quotations presented	Were participant quotations presented to illustrate the themes/findings? Was each quotation identified? e.g. participant number **YES**	Results, p 5-10
30. Data and findings consistent	Was there consistency between the data presented and the findings? **YES**	Results, p 5-10
31. Clarity of major themes	Were major themes clearly presented in the findings? **YES**	Results, p 5-10
32. Clarity of minor themes	Is there a description of diverse cases or discussion of minor themes?	Results p 5-10<br>Discussion p 6-12

Developed from: Tong A, Sainsbury P, Craig J. Consolidated criteria for reporting qualitative research (COREQ): a 32-item checklist for interviews and focus groups. International Journal for Quality in Health Care. 2007. Volume 19, Number 6: pp. 349 – 357
